# You don’t have to be rich to save money: On the relationship between objective versus subjective financial situation and having savings

**DOI:** 10.1371/journal.pone.0214396

**Published:** 2019-04-01

**Authors:** Dominika Maison, Marta Marchlewska, Katarzyna Sekścińska, Joanna Rudzinska-Wojciechowska, Filip Łozowski

**Affiliations:** 1 Faculty of Psychology, University of Warsaw, Warsaw, Poland; 2 Institute of Psychology, Polish Academy of Sciences, Warsaw, Poland; 3 Wroclaw Faculty of Psychology, SWPS University of Social Sciences and Humanities, Wroclaw, Poland; Ball State University, UNITED STATES

## Abstract

Saving is an important financial behavior that provides an individual with psychological security and boosts his/her overall sense of well-being. For this reason, scientists and practitioners have attempted to understand why some people save when others do not. One of the most common explanations for this phenomenon is that those individuals who earn more should be more willing to save their money. In line with this logic, people who have more money should be more likely to have savings. Considering the results of prior research, we expected objective financial situation (income) to be positively linked to having savings (i.e., propensity to have savings and the exact amount of savings). At the same time, however, we assumed that subjective financial situation (perception) should also be positively related to these variables. To test our assumptions, we conducted a nationwide representative survey (*N* = 1048) among Polish respondents, asking them about their objective and subjective financial situation. The results of a regression analysis showed that objective financial situation was indeed significantly positively related to having savings. However, subjective financial situation was also positively correlated with having savings (even when we controlled for objective financial situation and demographic variables). We discuss the implications of the links between objective versus subjective financial situations and having savings.

## Introduction

Even though there is no doubt that saving is an adaptive behavior, many people do not save at all. According to financial analyses, citizens in many European countries do not have sufficient savings to live a comfortable life without having to worry about fulfilling their basic needs. For example, a study conducted in 13 European countries (total sample size N = 13 936) by the ING Group (one of the world’s leading banking/financial companies) showed that 29% of Europeans declared that they had no savings, whereas 36% declared that the amount of their savings is equal to the amount of their living costs for three months [1]. The situation seems even more dramatic in the US, where according to a 2017 GoBankingRates survey, 57% of Americans have less than $1,000 in their savings accounts, and 39% have no savings at all [2]. This act of financial irresponsibility encourages economists and social scientists to search for individual predictors of saving behavior and ways to increase people’s willingness to save money.

The fact that some people have more while others have less in savings is most often explained by the amount of money people earn or have at their disposal [3–4]. For example, Davis and Schumm [5] investigated the relationship between saving behavior and household income and identified an income threshold below which respondents saved almost nothing. The positive relationship (r = .50) between annual family income and the amount of savings was only found among families that earned more than a certain amount. Other researchers state that income is the most important factor in determining saving behavior [6–7]. On the other hand, poor or low-income individuals are also able to save some money [8–12]. Moreover, a wealth of research has shown that the ability to put money aside is not only influenced by economic factors [13–15] but also by a range of psychological variables (see [16] for an overview). Considerable attention has been paid to the motives behind human decisions to start saving money [3, 17–21], as well as individual variables, such as risk aversion, locus of control or optimism [22–23]. Some studies also emphasize a positive relationship between time horizon and saving [24–26] and others stress the role of situational factors that might significantly impact a decision about whether to put money aside, for example, fear of death [27], feeling connected with one’s future self [28], feeling powerful [29], or feeling stressed [30]. Thus, the underpinnings of having savings may be much more complex than presented through most economic analyses and dependent on many other factors in addition to income. We propose that one of those factors might be a subjective financial situation, i.e., its perception.

### Subjective versus objective measures

Psychological literature stresses that in many life areas subjective assessment of a given situation is not always directly linked to objective facts. For example, research on life-satisfaction shows that it is only slightly related to one’s objective situation (i.e., income, health, family situation) especially when basic life needs are satisfied [31–32]. The literature also shows that, in some cases, the perception of a given situation has a stronger influence on decisions and behavior than objective facts. For example, the perception of body weight is more closely linked to internalizing and externalizing problem behavior, as well as social and thought problems, than an objective measure of body mass [33]. Underweight or overweight adolescents who consider themselves to be in a good shape have no more problems than those with a normal BMI. On the other hand, the perception of being ‘too thin’ and particularly ‘too heavy’ is a stronger predictor of problematic behaviors than actual weight, both among male and female adolescents. Another example was provided in the study conducted by Miron-Shatz Hanoch Doniger,Omer, and Ozanne [34], which showed that the willingness to pay for BRCA1/2 genetic testing (a positive test result is a substantial risk factor for breast and ovarian cancer) is affected by subjective but not objective numeracy (an ability to understand and manipulate numbers).

Similar patterns of results are also observed for variables reflecting one’s financial situation. In many cases, perception of financial situation or status had stronger predictive value for different behaviors than their objective measures. Firstly, subjective social status, understood as an individual perception of one’s own position in the social hierarchy [35], was shown to be a better predictor of need for utilization of health services than the ‘hard data’ [36]. Compared with objective indicators, subjective social status was more consistently and strongly related to psychological functioning and health related factors [37]. Moreover, subjective social status predicted health, even after accounting for traditional indicators of socioeconomic status, such as education, income, and occupation [38–40]. Secondly, perception of income inequality was demonstrated to be a more consistent predictor of morbidity and mortality than absolute income [41–42]. Finally, research on the relationship between one’s financial situation and life satisfaction showed a weaker correlation between life satisfaction and wealth (between .12 and .15 depending on the study) and a stronger correlation between life satisfaction and subjective financial situation (between .51 and .53 depending on the study) [43].

In the context of the abovementioned research results, a question emerges about the causal direction between well-being and financial behaviours. For many years researchers assumed that the better people’s lives are, the more their needs are satisfied, and the happier they should feel [44]. However, recently, researchers have begun to consider if this dependency may be based on a different direction–perception causing better life performance [45–46]. This so-called top-down approach (instead of bottom-up) assumes that happier people are not only more satisfied with specific aspects of their life but also perform better in different life areas [47–48]. In a longitudinal study, students who were more satisfied with life in their first year of university, turned out to have higher incomes than their less happy colleagues 19 years later [49]. In another study conducted by Lyubomirsky and colleagues [45], the direction of the relationship from happiness towards success (and not the other way around) was demonstrated in various areas of life and using various methodologies (cross-sectional, longitudinal, and experimental).

The abovementioned studies clearly demonstrate that one’s perception of a given situation should be taken into account next to objective indicators. Therefore, it is worth investigating whether incorporating one’s subjective financial situation next to objective measures would allow us to understand financial decisions better than analysing the objective measures alone. Moreover, those studies demonstrated that in many life areas a causal relation does not move from facts to perception but the other way around, from perception to performance, that is, from the subjective to objective level.

### Subjective financial situation

It is widely acknowledged that objective indicators of economic wealth might not always accurately reflect how people subjectively experience their financial situation [50–53]. Two people holding the same amount of wealth might perceive it differently, as their needs, aspirations, expectations or past experiences may be different [52, 54–59]. Moreover, subjective economic well-being might be affected by the level of assets and debt [60]. Two people with equivalent net worth but composed of different levels of assets and debt might perceive their financial situation as different in terms of their wealth. Specifically, people with a positive net worth feel wealthier when they have a lower level of debt and consequently fewer assets. On the other hand, people with a negative net worth feel wealthier when they have more assets despite having greater debt.

The subjective financial situation has been referred to as, among many other terms, perceived economic well-being [61], financial satisfaction [52], financial well-being [62], and economic strain [63]. Although the positive relationship between objective and subjective measures of one’s wealth is well established, its strength varies among studies, with correlation coefficients between the two ranging from values near .25 [64–66] to .50 [55, 67–68], with some even being nonsignificant [69]. Moreover, there are also people whose salaries are relatively high but who perceive their financial situation as bad and, in contrast, there are individuals who perceive their financial situation as extremely good even though it is objectively bad [51, 53, 70–73]. Some researchers term the latter case a *satisfaction paradox* [72, 74], which corresponds not only to financial satisfaction but also to general well-being. In a nationwide representative survey conducted in Poland [71], both objective and subjective measures of financial situation were introduced. The results showed that in some cases (among some people), there were significant inconsistencies between subjective and objective financial situations. Further analysis showed that this phenomenon involved two types of individuals: financial pessimists and financial optimists [71]. Financial optimists were those who had the lowest income but perceived their financial situation as good. Financial pessimists were those whose income was two times higher than the optimists’ salary but perceived their financial situation as bad. Comparisons of these two groups of people showed that financial pessimists were generally more prone to complain about their financial situation, feeling that there were many things they could not afford, and having more financial problems (e.g., debts) than the optimists.

The lack of a strong relationship between objective and subjective financial situation suggests that there may be certain individual difference variables that contribute to the process of evaluating one’s financial well-being. For example, it has been shown that one’s attitudes toward money might affect the perception of one’s financial situation [54, 75]. The process of social comparisons, especially regarding how people perceive their income in comparison to others, also plays an important role in the perception of own finances [76].

It has also been demonstrated that financial satisfaction is linked to a whole array of financial behaviors and characteristics of the consumer. Xiao, Chen, & Chen [77] examined the relationships between financial satisfaction and consumer financial capability (i.e., perceived financial capability, financial knowledge, and financial behavior) and demonstrated that financial satisfaction was positively associated with perceived financial capability, desirable financial behaviors and subjective financial knowledge. Additionally, financial satisfaction decreased risky financial behaviors. In another study [78], the association between financial satisfaction and the propensity to plan for long-term goals was examined and it was demonstrated that financial satisfaction, after controlling for socio-economic and other financial capability factors, made unique contributions to the propensity to plan for long-term goals. Moreover, financial satisfaction was also demonstrated to be positively associated with preparing a household financial budget [60, 79].

The subjective financial situation impacts various domains of life. It is believed to be a subconstruct of general well-being [52] and was recently shown to influence investment decisions. A study conducted by Sekścińska, Rudzińska-Wojciechowska and Maison [80] revealed that male participants who perceived their financial situation as good (vs. bad) were more prone to invest. At the same time, their objective financial situation did not affect the propensity to invest.

### Hypotheses

Taking into account the abovementioned role of subjective financial situation in different areas of life decisions, we assumed that in regard to explaining having savings, subjective financial situation should be at least as important as objective financial situation or, in some cases, even more important. To test this assumption, we conducted a study using a large, heterogeneous sample. Participants’ objective (income) and subjective (perception of) financial situation were measured. Bearing in mind that there are people who do save regularly, but their amount of savings is low due to their low income, we decided to include two measures of having savings: whether one has savings or not and the amount of savings. In other words, we introduced two measures of having savings to differentiate between: (1) the amount of savings, which is a traditional indicator of saving practices, and (2) the propensity to have savings–(i.e., whether one has savings or not) which can be understood as a skill or a tendency to save. We predicted that both objective and subjective financial situations should be positively related to one’s propensity to have savings and the amount of money saved.

Based on the research reviewed above, which indicates that the objective measures may not accurately reflect how people experience their financial situation, we formulated the following hypotheses:

H1. Objective financial situation would be positively related to the propensity to have savings (dichotomous variable) and the amount of savings.

H2. Subjective financial situation would be positively related to the propensity to have savings (dichotomous variable) and the amount of savings even when controlled for the variance shared with objective financial situation and demographic variables.

H3. The effects of objective financial situation on the amount of savings, which is strictly related to the specific amount of money, should be stronger than the effects of subjective financial situation.

H4. The effects of subjective financial situation on the propensity to have savings, which is largely related to the mere intention to save money, should be stronger than the effects of objective financial situation.

## Method

### Participants and procedure

The study was conducted via an internet research panel on a nation-wide sample. The participants were randomly selected from the panel users, and demographic structure of the sample was controlled in order to make it compatible with the structure of the Polish population. The quotas were selected based on the distribution of gender, age, education and size of town in the population of Poles. The sample consisted of 1048 respondents (550 women) between the ages of 18 and 69 (*M*_*age*_ = 42.96, *SD* = 14.82). Several measures and scales were presented to participants, including measures of subjective and objective financial situations, questions about propensity to have savings, amount of money saved and demographics (e.g., gender, age).

All participants provided their informed consent to take part in the research after reading detailed information about the study. Participants were asked to click on a link to the study if they agreed to take part in the research. Otherwise, they did not participate in the study. All participants of the panel are rewarded for their participation in every study with points, which can be exchanged for rewards of their choice. Each participant received exactly the same amount of points as for his/her participation in the study.

### Measures

Two aspects of financial situation were measured. The first one was objective financial situation–reflected by the amount of monthly income of the household–and the second one was subjective financial situation measured in two ways, as a general evaluation of financial situation and as an assessment of household purchasing power.

#### The objective financial situation

The objective financial situation (variable: *objective FS*) was measured using one question: “Please indicate the average monthly net income of your household”. To avoid numerous missing values in the database, the answers to the question were given on a 7-point scale from *1 = less than 1000 PLN* to *7 = more than 10*,*000 PLN*.

#### The subjective financial situation

The subjective financial situation was measured with two questions. In the question about general subjective financial situation (variable: *subjective FS–general*), participants were asked to assess their material situation on a 7-point scale (1 = *very bad* to 7 = *very good*). In the question about the subjective household’s purchasing power (variable: *subjective FS–household purchasing power*), the participants were asked to indicate which out of five statements best describes their household financial situation, ranging from 1 = *We do not have enough money for the most urgent needs* to 5 = *We have enough money*, *we do not have to save even for bigger expenses*.

Having savings was measured by two different variables: in the traditional way, as an amount of savings held by the respondent, and as a propensity to have savings understood as having any savings, regardless of the amount of savings.

#### Amount of savings

Amount of savings was measured as declared amount of savings the person has. Respondents indicated amount of savings using 10 intervals: from 1 *= no savings* to 10 *= more than 100*,*000 PLN* (2 = less than 500 PLN; 3 = 501–1000 PLN; 4 = 1001–5000 PLN; 5 = 5001–10 000 PLN; 6 = 10 001–20 000 PLN; 7 = 20 001–30 000 PLN; 8 = 30 000–50 000 PLN; 9–50 001–100 000 PLN; 10-more than 100 000 PLN).

#### Propensity to have savings

Propensity to have savings was measured with one question: *Do you have any savings*? The answer was coded as 1 = Yes or 0 = No.

Choosing these measures of objective and subjective financial situation, we were conscious of their limitations, especially that they might not exactly capture the same phenomenon. Objective financial situation referred only to income, but by asking a question about subjective financial situation we didn’t have control over what respondents could have in mind when answering this question (they might only consider income or also other things, e.g., properties). Nevertheless, we chose this form of question in order to entrench their simplicity.

## Results

### Zero-order correlations

Zero-order correlations between variables and scale properties are presented in Table 1. We found significant positive relationships between objective financial situation and (1) propensity to have savings and (2) amount of savings, which is in line with previous research linking objective financial situation (e.g., income) to saving [3–4]. Both indicators of subjective financial situation were also positively related to propensity to have savings and amount of savings. Objective financial situation was significantly positively related to both indicators of subjective financial situation (general and purchasing power).

**Table 1:** Correlations and descriptive statistics.

Measure	1.	2.	3.	4.
1. Objective financial situation *Scale from 1 to 7*				
2. Subjective financial situation: general *Scale from 1 to 7*	.37 *p* < .001			
3. Subjective financial situation: purchasing power *Scale from 1 to 5*	.46 *p* < .001	.59 *p* < .001		
4. Saving: amount of savings *Scale from 1 to 10*	.40 *p* < .001	.38 *p* < .001	.43 *p* < .001	
5. Saving: propensity to have savings *1 = yes; 0 = no*	.27 *p* < .001	.39 *p* < .001	.41 *p* < .001	.80 *p* < .001

### Regression analyses

We performed a stepwise multiple regression analysis to investigate the relationships between financial situation measures (objective vs. subjective) and having savings (amount of savings or propensity to have savings). Moreover, we decided to test interaction effects between objective and subjective measures of financial situations on having savings, and thus, financial situation variables were mean-centered prior to the analyses. In both analyses, we also controlled for basic demographics (age and gender).

#### Saving: Amount of savings as an outcome variable

First, we conducted a hierarchical multiple regression analysis to test the hypothesis that subjective financial situation (general and subjective household purchasing power) would be more strongly related to the amount of savings than objective financial situation (Table 2).

**Table 2:** Predictors of savings: Amount of savings.

	Step 1				Step 2				Step 3			
Variables	*B*	*SE*		*p*	*B*	*SE*		*p*	*B*	*SE*		*p*
Gender	-0.22	0.18		.22	-0.28	0.17		.10	-0.22	0.17		.18
Age	0.02	0.01		.001	0.03	0.01		< .001	0.03	0.01		< .001
Objective FS	0.79	0.07		< .001	0.38	0.07		< .001	-0.13	0.24		.58
Subjective FS: general					0.37	0.08		< .001	0.40	0.08		< .001
Subjective FS: purchasing power Objective FS × Subjective FS: general					0.91	0.13		< .001	0.93 0.10	0.13 0.05		< .001 .05
Objective FS × Subjective FS: purchasing power									0.16	0.08		.04
*F*	55.74		< .001		68.26		< .001		52.83		< .001	
*R*^*2*^	.17				.30				.32			

In Step 1, we introduced objective financial situation and demographics (gender, age). We found significant positive effects of age and objective financial situation on amount of savings.

In Step 2, we introduced variables coding subjective financial situation: general and purchasing power, and found their positive effects on amount of savings. After introducing subjective financial situation variables, we still found a significant (albeit weaker) effect of objective financial situation and a significant effect of age.

**Fig 1:**
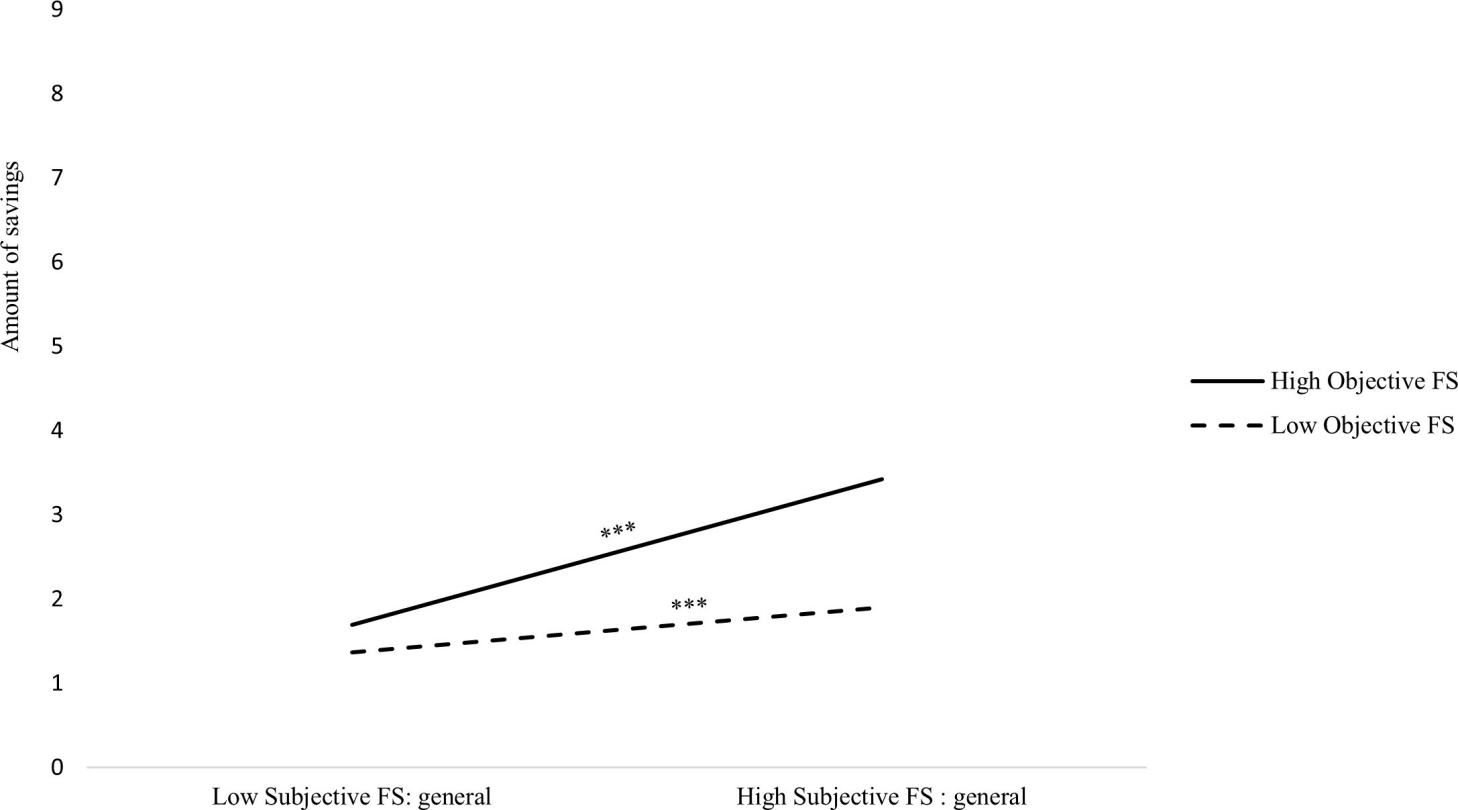
Interaction effect of objective financial situation and subjective financial situation (general) on saving: Amount of savings. *** *p* < .001.

**Fig 2:**
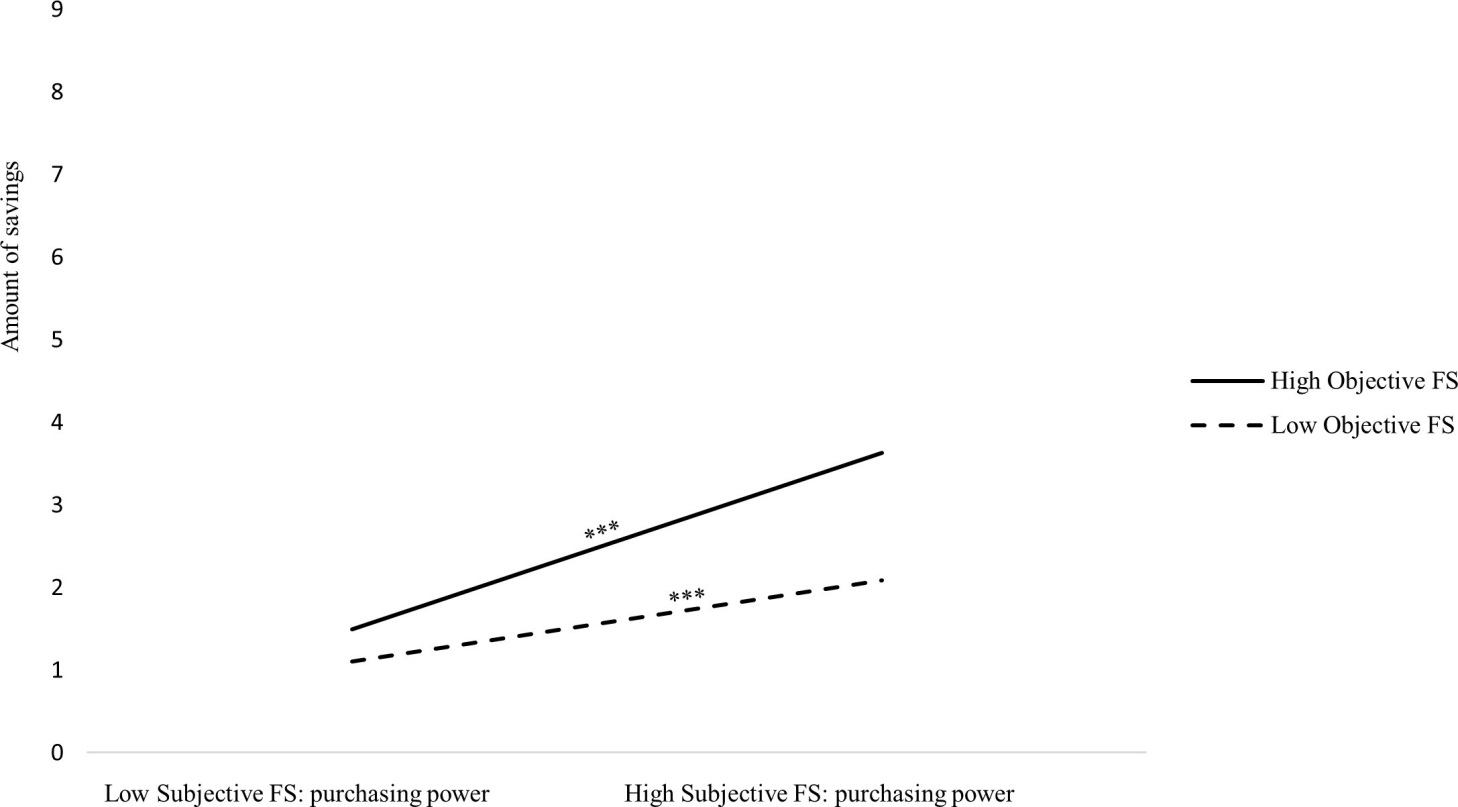
Interaction effect of objective financial situation and subjective financial situation (perception of household purchasing power) on saving: Amount of savings. *** *p* < .001.

In Step 3, we introduced two two-way interactions between objective financial situation and (a) subjective financial situation: general and (b) subjective financial situation: perception of household purchasing power. We found significant positive effects of subjective financial situation (general and perception of household purchasing power) on amount of savings. We also found a significant positive effect of age. However, we found no significant effect of objective financial situation on amount of savings and a marginally significant interaction between objective financial situation and subjective financial situation: general. Simple slope analysis indicated that among people low in subjective financial situation (general), the effect of objective financial situation was positive but not significant, *B* = 0.11, *SE* = 0.10, *p* = .21 and was positive and significant among people high in subjective financial situation (general), *B* = 0.56, *SE* = 0.08, *p* < .001 (Fig 1). Moreover, we also found a similar significant interaction between objective financial situation and subjective financial situation (perception of household purchasing power). Again, simple slope analysis indicated that among people low in subjective financial situation (perception of low purchasing power of the household), the effect of objective financial situation was positive but not significant, *B* = 0.14, *SE* = 0.09, *p* = .11, but was positive and significant among people high in subjective financial situation (perception of high purchasing power of the household), *B* = 0.57, *SE* = 0.08, *p* < .001 (Fig 2).

#### Saving: Propensity to have savings as an outcome variable

Second, we conducted a stepwise logistic binominal regression analysis to investigate the relationships between financial situation measures (objective vs. subjective) and the propensity to have savings. Moreover, we decided to test interaction effects between objective and subjective measures of financial situations on having savings, and thus, financial situation variables were mean-centered prior to the analyses. In both analyses, we also controlled for basic demographics (Table 3).

**Table 3:** Predictors of savings: Propensity to have savings.

	Step 1			Step 2			Step 3		
Variables	*B*(*SE*)	*OR*	*p*	*B*(*SE*)	*OR*	*p*	*B*(*SE*)	*OR*	*p*
Gender	-0.05 (0.14)	0.95	.73	-0.10(0.16)	0.91	.53	-0.10(0.16)	0.91	.55
Age	-0.01(0.01)	1.00	.33	0.004(0.01)	1.004	.46	0.004(0.01)	1.00	.45
Objective FS	0.42 (0.06)	1.53	< .001	0.11(0.07)	1.12	.08	0.13(0.07)	1.13	.06
Subjective FS: general				0.46(0.08)	1.58	< .001	0.44(0.08)	1.56	< .001
Subjective FS: purchasing power Objective FS × Subjective FS: general				0.83(0.13)	2.29	< .001	0.84(0.13) -0.07(0.05)	2.32 0.94	< .001 .21
Objective FS × Subjective FS: purchasing power							0.09(0.09)	1.09	.32
2 log-likelihood	1112.22			963.71			961.65		
Nagelkerke’s *R*^*2*^	.10			.30			.30		

In Step 1, we introduced objective financial situation and demographics (gender, age). We found a significant positive effect of objective financial situation and no significant effects of age or gender on propensity to have savings.

In Step 2, we introduced variables coding subjective financial situation: general and perception of household purchasing power and found their positive effects on propensity to have savings. After introducing subjective financial situation variables, the effect of objective financial situation on propensity to have savings became only marginally significant.

In Step 3, we introduced two two-way interactions between objective financial situation and (a) subjective financial situation: general and (b) subjective financial situation: perception of household purchasing power; none proved to be significant. However, we still found a significant positive effect of subjective financial situation (general and perception of household purchasing power) on propensity to have savings.

## Discussion

In this research, we investigated the role of objective (i.e., income) and subjective (i.e., perception of) financial situation in having savings. We conducted a study on a nation-wide sample that reflected the demographic structure of the Polish population. The results of the study confirmed our assumptions and showed that both objective and subjective financial situations are important predictors of having savings. However, the positive link between objective financial situation and having savings became weaker (DV: amount of savings) or insignificant (DV: propensity to have savings) when subjective financial situation was accounted for.

In line with previous findings [22, 69, 81–87], an objective financial situation was positively linked to the amount of money individuals saved. Even after introducing subjective financial situation, the effect of objective financial situation on the amount of savings was still significant, although slightly weaker. Nevertheless, the interaction between subjective and objective financial situation was also significant: specifically, we found that the objective financial situation was only significantly positively related to the amount of savings among those people who had high scores on subjective financial situation. Thus, the results showed that subjective financial situation is a very important factor related to the amount of money people save. Thus, it is possible that when one earns more money but perceives his/her financial condition as rather weak, he/she would not necessarily be more likely to have savings than those who earn much less.

The same pattern of results was observed irrespective of the method of measuring subjective financial situation, either in general or as perception of household purchasing power. These results shed new light on previous findings, which mainly focused on the positive relationship between the objective financial situation and the amount of savings. Although objective financial situation was significantly positively related to the amount of savings, this was especially the case among participants with high scores on subjective financial situation. Thus, those who have more money at their disposal have more savings, but only as long as their perception of their financial situation is good. These findings can be partly explained by Bandura’s self-efficacy theory [88] according to which there are people more (vs. less) prone to believe that they have the ability to influence their lives and, thus, achieve their goals. Previous research [89] showed that self-efficacy is positively related to optimism. Thus, it is possible that individuals high in self-efficacy who believe they have the ability to influence the events of their own lives would also be more prone to be financial optimists and perceive their financial situation as relatively better than those who score low on self-efficacy scales (i.e., financial pessimists). This mechanism can further lead to different financial decisions (also related to having savings). Such positive perceptions of one’s abilities in the financial domain may in fact lead to a better perception of one’s financial situation and, as a result, evoke saving behavior. Still, further empirical investigation is needed to test these assumptions.

We found a similar pattern of results when analyzing whether a participant has any money saved independently of the amount of money saved (propensity to have savings) as a dependent variable. These results show a similar pattern, although they are stronger, and their implications are slightly different. The first step of the analysis showed a positive relationship between objective financial situation and propensity to have savings (similarly to the results when amount of savings was the dependent variable). However, after we introduced subjective financial situation into the equation, we found a significant effect of subjective financial situation on propensity to have savings, whereas the effect of objective financial situation was no longer significant. This result means that subjective financial situation is strongly linked to the propensity to have savings. It also means that whether people have any savings or not might be independent of the amount of money they earn. If someone has very little money at his/her disposal but has a very high propensity to have savings, it is very possible that he or she will have some money put aside. An important consequence of this characteristic is that if someone has the propensity to have savings, and their income rises, his/her savings will also rise. However, if someone has no propensity to have savings, regardless of the amount of money earned or obtained from other sources (e.g., inheritance or a lottery win), he/she will probably have no savings.

Our study clearly demonstrated that objective and subjective financial situation are significantly, though not strongly, related to each other. Thus, it seems crucial to account for not only objective but also subjective financial situation when analyzing financial behaviors. In some cases, for example, propensity to have savings, perceptions can take on an even greater importance than objective measures.

### Limitations and further research

Although the present study is based on a large, heterogeneous sample and brought several interesting results, it has some disadvantages and limitations. Firstly, we relied solely on self-reported data. Therefore, the present study has all the limitations that are characteristic for self-report measurements. Secondly, as the study was based on cross-sectional data, no assumptions of causality can be drawn from the results. Although it is probable that subjective financial situation provides bases for financial decisions, it is also possible that a reassuring awareness of having some money put aside in case of a rainy day impacts one’s perception of one’s financial situation. It would then be highly desirable to apply an experimental design in future studies to establish the direction of the described relationship. Moreover, the study was focused on one aspect of saving practices–it investigated whether one has some money put aside for the future and, if so, how much it is. We did not control where the money came from, specifically whether it was actively accumulated or, for example, inherited or won in the lottery. However, regardless of the source of the money, the fact that it is perceived as ‘savings’ means that the consumer is prone to put and keep money aside rather than consume all the available resources. Nevertheless, further studies are needed to investigate how one’s subjective wealth is linked to other saving practices. For example, they could take into account the strategies that consumers use in order to accumulate savings, saving motives or saving horizon.

Finally, one might argue that the subjective and objective measures of participants’ financial situations are not parallel and not focused on similar aspects of one’s wealth, as the objective measure captures only information about participants’ income, whereas subjective assessment also captures information about assets and a relation between income and expenses. It is possible that subjective measures reflect more information than objective ones, as participants take into account large amounts of data when answering a single question about the perception of their finances. Thus, future research would do well to measure objective financial situation in a more developed and precise manner, for example, by asking about different dimensions of this phenomenon (i.e., going beyond income and focusing on a broader aspect of financial assets). Also, when it comes to methodological improvements, some of the variables in our study (e.g., the amount of savings) were measured with the use of an interval scale. Future work would do well to measure similar variables by asking about the exact amount of money (earned or saved).

Despite the acknowledged limitations, the present study opens several avenues for further research. Apart from the directions indicated above, a dynamic nature of individual financial circumstances should be taken into account. Repeated measures of subjective wealth over the course of life will enhance the understanding of determinants of saving decisions. Moreover, there is a need to verify to what extent the perception of finances is related to other financial decisions, such as spending, borrowing, insuring or investing. Finally, when planning further research on the *satisfaction paradox*, it would be worth considering the results of research [71] that has shown that there are two types of consumers in relation to their finances: financial pessimists and financial optimists. Future research might investigate the differences in attitudes and saving behaviors of these two groups.

### Practical implications

In the context of saving behavior, a very important question is how to increase the amount of savings in society. Many studies based on declarations provide results that are, to some extent, misleading, indicating that saving behavior can be obtained directly by increasing the wealth of a society. However, our study suggests that augmenting saving in society could probably be achieved more indirectly by influencing individuals’ positive perceptions of his/her financial situation. Such an indirect effect can be achieved, for example, by mental training related to perceptions of one’s financial situation. This possibility is an important conclusion for financial counselors who work with people to increase financial well-being.

The results of the described study also have more general implications related to marketing research. In the majority of marketing strategies, target groups for products are usually defined by level of income, assuming that people with a higher income will use more expensive products or prefer more luxurious brands than people with a lower income. The results of our study suggest that subjective financial situation can be at least as important a factor in explaining what people do with their money as objective measures.
